# Is It Necessary to Adapt Training According to the Menstrual Cycle? Influence of Contraception and Physical Fitness Variables

**DOI:** 10.3390/life13081764

**Published:** 2023-08-17

**Authors:** Paula Recacha-Ponce, Eladio Collado-Boira, Pilar Suarez-Alcazar, Macarena Montesinos-Ruiz, Carlos Hernando-Domingo

**Affiliations:** Faculty of Health Sciences, Jaime I University, 12071 Castello de la Plana, Spain; recacha@uji.es (P.R.-P.); malcazar@uji.es (P.S.-A.); macarenamontesinosruiz@gmail.com (M.M.-R.); hernando@uji.es (C.H.-D.)

**Keywords:** menstrual cycle, contraceptive cycling, $\dot{V}$O_2_max; physical fitness; sensory threshold

## Abstract

(1) Background: The influence of the menstrual cycle on physical fitness in athletes is controversial in the scientific literature. There is a marked fluctuation of sex hormones at three key points of the menstrual cycle, where estrogen and progesterone vary significantly. Hormonal contraception induces hormonal levels different from the natural menstrual cycle, requiring specific study in relation to physical fitness. (2) Method: Women aged 18 to 40 years with regular natural menstrual cycles and women using hormonal contraception were recruited, creating two study groups. All participants needed to be athletes classified as level II–III, based on training volume/physical activity metrics, among other variables. To assess their physical fitness, cardiorespiratory fitness (measured by $\dot{V}$O_2_max), high-speed strength, hand grip strength, and flexibility were evaluated. Blood samples were taken to determine the menstrual cycle phase through analysis of sex hormone levels. Additionally, urine tests for ovulation detection were performed for the natural menstrual cycle group. Neurosensory stimulation tests were incorporated to measure sensory thresholds and pain thresholds in each phase. Body composition in each phase and its relationship with the other variables were also taken into account. (3) Results: Athletes in the natural cycling group showed differences in $\dot{V}$O_2_max (mL·kg^−1^·min^−1^) (phase I = 41.75 vs. phase II = 43.85 and (*p* = 0.004) and phase I vs. phase III = 43.25 mL·kg^−1^·min^−1^ (*p* = 0.043)), as well as in body weight (phase I = 63.23 vs. phase III = 62.48 kg; *p* = 0.006), first pain threshold (phase I = 1.34 vs. phase II = 1.69 (*p* = 0.027) and phase III = 1.59 mA (*p* = 0.011)), and sensitive threshold (phase I = 0.64 vs. phase II = 0.76 mA (*p* = 0.017)). The pain threshold was found to be an important covariate in relation to $\dot{V}$O_2_max, explaining 31.9% of the variance in phase I (*p* = 0.006). These findings were not observed between the two phases of contraceptive cycling. (4) Conclusion: The natural menstrual cycle will cause significant changes in the physical fitness of athletes. The use of hormonal contraception is not innocuous. Women with natural cycles show an increase in cardiorespiratory fitness in phases II and III, which is a factor to be considered in relation to training level and workload.

## 1. Introduction

Female sex hormones are the main regulators of fertility and reproduction with secretion that varies throughout the ovarian cycle and have numerous physiological effects of a systemic nature [1]. Given the diverse effects of estrogen and progesterone, it would not be surprising for the timing of the menstrual cycle (MC) to influence the physical fitness of female athletes. Aspects such as cardiorespiratory capacity, high-speed strength, hand grip strength, and flexibility [2] could be modified due to the hormonal fluctuations associated with the menstrual cycle. These hormonal fluctuations are also responsible for the physical symptoms related to the menstrual cycle, such as pain [3], which could impact physical fitness to a greater or lesser extent. Objective measurement of these symptoms could contribute to addressing this question. However, several meta-analyses have concluded that studies with higher methodological quality are needed to determine the effects of sex hormones on physical fitness [4,5]. Even if these changes were minimal, they would be of importance in female athletes, where the goal is to achieve the best performance in competitive events [6].

There are studies confirming that hormones related to the menstrual cycle influence cardiovascular, respiratory, neuromuscular, neurocognitive, and metabolic parameters, and consequently, physical fitness [7,8,9,10]. Conversely, there are also studies in the literature that contradict the previously mentioned findings, concluding that the menstrual cycle does not influence physical fitness [11,12,13]. This discrepancy could be attributed to the hormonal patterns of natural menstrual cycles. It is possible that results have been generalized among women with natural ovulatory cycles and women with deficient luteal phases, where progesterone levels are not sufficient to trigger ovulation [14], leading to an error as these women have different hormonal patterns. In physically active women, this becomes particularly important as they have a higher risk of experiencing deficient luteal phases [15], with reported rates of up to 30% among women engaging in exercise [16]. Therefore, it is crucial to study this specific profile without generalizing the results to women who do have physiological luteal phases. Determining sex hormones (estrogen and progesterone) in blood is recommended as the gold standard for determining menstrual cycle phases and differentiating between ovulatory cycles and cycles with deficient luteal phases [14,17]. The measurement of the free androgens index (FAI) is also important. FAI measures total testosterone and sex hormone-binding globulin (SHBG). FAI measurement helps determine whether a cycle is ovulatory or not, as it is elevated in anovulatory cycles [16,18]. However, testosterone levels alone are similar between ovulatory cycles [19].

Furthermore, another important profile to consider is hormonal contraception, as it differs from the natural menstrual cycle and requires specific analysis [20,21,22,23]. These provide constant levels of exogenous estrogens and progestogens, suppressing ovulation [24]. Hormonal contraceptives can reduce premenstrual syndrome, menstrual pain, blood loss, and iron deficiency [25,26]. As they offer stable hormonal environments, they are sometimes used by athletes to predict or suppress their menstruation [27,28]. However, these may not be without consequences, as various studies have concluded that they could impact variables influencing physical fitness [21,22].

More prospective and randomized clinical trials are needed in trained athletes, utilizing precise hormonal measurements to verify menstrual cycle phases, in order to investigate the short- and long-term effects of both the natural menstrual cycle phases and hormonal contraceptives on female athletes [24].

The aim of this study was to investigate whether the menstrual cycle, both natural menstrual cycling (MC) and contraceptive MC, influences variables related to physical fitness, body composition, pain, and sensitivity.

## 2. Materials and Methods

Experimental design. This is a study with a cross-sectional prospective cohort design. Two study groups were formed: the natural cycling group and the contraceptive cycling group. Assessments were conducted targeting the physical fitness based on the athlete’s menstrual cycle phase.

**Figure 1:**
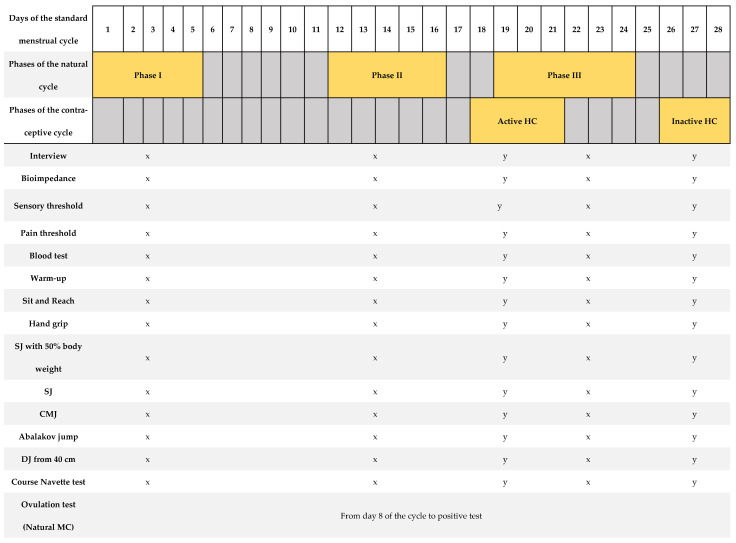
Summary of the research protocol, based on a menstrual cycle of 28 days’ duration [12].

Figure 1 shows a summary of the protocol for the tests performed. Each test described below was performed three times for the natural cycling group and twice for the contraceptive cycling group, depending on the phase of the participant’s MC. The timing of assessments was based on the menstrual cycle’s phases where the greatest hormonal fluctuations occur [17,23]. Each of these visits was adjusted to the standardized test protocol, which included verification of the athlete’s health, measurement of body composition, measurement of the pain and sensory threshold, extraction of blood sample, standardized warm-up, and performance of the 8 tests to evaluate physical performance (tests to measure the level of flexibility, high-speed strength, hand grip strength, and cardiorespiratory fitness). The procedures carried out were identical among all participants and at all visits. All participants began the study in phase I, followed by phase II and phase III (natural MC group). For the contraceptive MC group, the study commenced in the inactive phase and concluded with the active phase. All athletes were instructed and familiarized with all tests performed, conducting several trial attempts prior to valid attempts. It should be noted that the participants are trained women and knowledgeable about all these tests in their usual training. During all tests, participants received standardized verbal instructions from the research team.

We provide overview of the study protocol describing days of assessments and the outcomes measured in relation to a standard 28-day menstrual cycle. The highlighted days correspond to the range of cycle days where measurements were taken individually for each woman. The testing period included three visits, each following a standardized testing protocol as outlined: a brief medical history before proceeding with the rest of the tests, bioimpedance analysis, measurement of sensory threshold and first pain threshold, blood extraction, standardized warm-up, and a battery of physical tests: sit and reach, hand grip, squat jump with 50% body weight, squat jump, counter movement jump, Abalakov jump, and drop jump from a height of 40 cm. A urinary ovulation test was performed each morning from day 8 of the MC until a positive test result was observed. SJ: squat jump; CMJ: counter movement jump; DJ: drop jump. x = tests carried out in the natural MC group; y = tests carried out in the contraceptive MC group.

### 2.1. Determination of the Phases of the Natural MC

The hormonal fluctuations are most noticeable in three key phases of the natural MC [17]. Following the recommendations of Jonge XJ et al. (2019), this study took into account the following phases, listed in the order of appearance in a eumenorrheic MC:

- Phase I: Where estrogen and progesterone have low concentrations. In this research, this phase was considered from the first day of bleeding to day 5 [17,23]. The assessment of phase I was conducted as soon as possible from the first day of bleeding, with the cutoff for this first assessment being day 5 of the menstrual cycle.
- Phase II: Detected by a positive urinary ovulation kit (Luteinizing Hormone (LH) screening). It is characterized by a marked increase in estrogen, as well as progesterone (compared to phase I). This study considered this phase from a positive urinary ovulation kit until 48 h after detection [23].
- Phase III: Both estrogen and progesterone have higher levels than in the previous phases. Progesterone had to be greater than 16 nmol·L^−1^ [17]. This study considered this phase 7 days after detecting the positive urinary ovulation [17,23]

The verification of these phases was determined taking into account the following recommendations from the literature:

- Menstrual cycle mapping: Women communicated with the research team on the first day of their menstruation, which was considered as day 1 of the cycle.
- Urinary LH measurement: Participants performed the ovulation detection test from day 8 of the cycle until a positive result was observed on the urine strip (Ovulation test, Acofar, Madrid, Spain). It was requested that it should be carried out first thing in the morning, and an appointment was scheduled as soon as possible to carry out the tests. During the first visit, women in the natural MC group were instructed on the use of urinary LH detection strips. For those not obtaining a positive result, tests were repeated in the next cycle. A negative test result on two consecutive occasions led to the exclusion of the athlete from the study. The result was verified by photographic evidence [17,23].
- Serum hormone analysis: Estrogen and progesterone levels were determined. In addition, LH and follicle stimulating hormone (FSH) were also measured in each phase, in order to detect possible pathologies such as polycystic ovary syndrome (PCOS). To determine whether ovulation had occurred or not, a minimum limit of 16 nmol·L^−1^ of serum progesterone was stated [17,23], determined between 7 and 9 days after a positive ovulation was detected in the urine. Subsequently, based on the levels of progesterone obtained in the analysis, all those who showed serum levels of progesterone greater than 16 nmol·L^−1^ were included in the group of women with natural ovulatory cycles. A lower result was grounds for exclusion from the analysis [17,23].

In this study, following the outlined guidelines, the assessments for women with a natural menstrual cycling were conducted at the following intervals: the first visit (between day 1 and 5 of the cycle), the second visit (between day 12 and day 16), and the third visit (between day 19 and day 24 of the cycle).

### 2.2. Phases of the Contraceptive MC

In the case of women with a contraceptive cycle, they were analyzed twice. The period of active contraceptive use, characterized by elevated exogenous hormone levels (between days 18 and 21 of the cycle), and the period of inactive contraceptive use, characterized by elevated endogenous hormone levels (between days 26 and 28 of the cycle), were both analyzed. In monophasic cycles, the exogenous concentration of ethinyl estradiol doubles from day one of active contraceptive use until day 21 [29,30]. The progestin concentration triples from day 1 to days 8–11 and remains stable thereafter until day 21 [30,31]. All types of hormonal contraception (HC) used were monophasic with low doses of ethinyl estradiol (<0.03 mg) and second- or third-generation progestogens (levonogestrel, etonogestrel, drospirenona) [32].

All women initiated the study at the end of the phase of the contraceptive cycle (between days 26 and 28 of the cycle, when endogenous hormone levels are at their peak). The second and final visit occurred during the late stage of the active phase of the contraceptive cycle (between days 18 and 21 of the cycle, when exogenous hormone levels are at their peak).

#### 2.2.1. Study Variables

- Hormonal variables: progesterone (nmol·L^−1^), estrogen (pmol/L), FSH (mIU/mL), LH (mIU/mL), total testosterone (nmol/L), SHBG (nmol/L), urinary LH (only in natural cycle), progesterone/estrogen ratio (P/E ratio), and FAI.
- Variables related to physical fitness:
  ○ Cardiorespiratory fitness: $\dot{V}$O_2_max (mL·kg^−1^·min^−1^) (indirectly calculated using the Course Navette test formula, explained later) and total meters achieved in the Course Navette test (m).
  ○ High-speed strength: squat jump 50% additional body weight (cm), squat jump (cm), counter movement jump (cm), Abalakov jump (cm), and drop jump from 40 cm (cm).
  ○ Hand grip strength: dominant hand grip (kg).
  ○ Flexibility: sit and reach test (cm).
- Variables related to body composition: height, body mass (kg), total body water (L), body fat percentage (%), skeletal muscle mass (kg), BMI (kg·m^−2^).
- Variables related to sensitivity and pain: sensory threshold (mA), first pain threshold (mA).

#### 2.2.2. Participants

Women with natural menstrual cycling and women with contraceptive menstrual cycling were recruited. All women were classified within levels II and III of the classification framework by McKay et al. [33], based on training volume and performance metrics. The participants were recruited through social networks with the dissemination of the research. Women interested in participating were informed and provided with a questionnaire through the Qualtrics^®^ platform. Participants were selected based on the established inclusion and exclusion criteria.

The inclusion criteria for both groups were as follows: (1) healthy women, (2) age between 18 and 40 years, (3) body mass index (BMI) ≥ 18.5, (4) being classified within level II and level III of the classification framework by McKay et al., and (5) non-consumption of alcohol or tobacco.

Specifically for the natural MC group: women with regular menstrual cycles lasting between 25 and 35 days for the past 6 months.

Specifically for the contraceptive MC group: women who have been using hormonal contraception for at least 6 months, with monophasic cycles, and low-dose ethinyl estradiol (<0.03) and second- or third-generation progestogens.

The sociodemographic characteristics are defined in Table 1.

**Figure 2:**
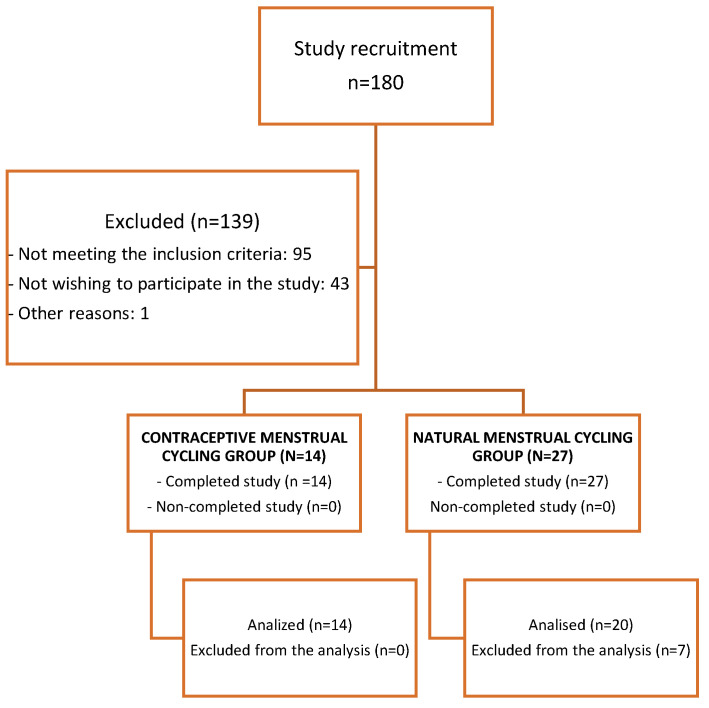
Flowchart to recruitment.

The flowchart related to recruitment and the final number of participating athletes can be observed in Figure 2.

The study was conducted in accordance with the Helsinki Declaration, approved by the local ethical committee of Jaume I University (CD/77/2020). All participants provided written consent before participating in the study. The study was registered at Clinical.Trials.gov (ID: NCT05576740).

#### 2.2.3. Test protocol

Figure 1 shows a summary of the protocol for the tests performed.

The order of the tests was the following for all participants in both study groups:

I. Interview
II. Bioimpedance
III. Sensory and pain threshold
IV. Blood test
V. Warm-up and fitness level assessment tests:
  - Flexibility: sit and reach test
  - Hand grip strength: maximum grip dominant hand
  - High-speed strength: squat jump with 50% body weight, squat jump, countermovement jump, Abalakov jump, and drop jump from 40 cm
  - Cardiorespiratory fitness: Course Navette test

(I) Interview

Following the established test protocol, potential symptomatology/acute illness, medication intake, and the timing for the last intake were initially verified during the first visit. For HC users, it was verified that the administration method had been carried out correctly. The timing of the tests, as well as previous exercise, was adapted according to the participant’s work and sports calendars, ensuring that the 2 visits (contraceptive MC) or 3 visits (natural MC) occurred at the same time of day and with the same preconditions.

(II) Bioimpedance

Next, the sizing and bioimpedance measurements were performed. Height was measured using a SECA 213 portable stadiometer (Seca GmbH & Co. Kg, Hamburg, Germany). To obtain the body mass index (BMI), percentage of fat mass (%FM), percentage of lean body mass (%LBM), skeletal muscle mass (kg), and percentage of water (%H_2_O), a bioelectrical impedance scale (Tanita BC-780MA, Tanita Corp., Tokyo, Japan) was used [34]. The measurements were made following the manufacturer’s guidelines. The skin and electrodes of the scale were impregnated with conductive gel before the test.

Additionally, following the recommendations for performing bioimpedance, the athlete should not have consumed any food or beverages at least 2.5 h prior to the assessment [34].

(III) Sensory and first pain threshold test

For the sensory and pain measurement tests, a high-voltage stimulator (DS7A, Digitimer Ltd., Welwyn Garden City, UK) was employed [35] to deliver a series of percutaneous electrical impulses with gradually increasing amplitude through electrodes placed in the flexor muscle of the wrist. Participants were instructed to report any sensations associated with the current, in response to the increase in power. The first sensation was identified was defined as the sensory threshold (ST). The participants finished this part of the test when they detected the first electrical impulse. The intensity was then increased until the first sensation of pain was observed [36,37]. After reporting the first painful sensation, the intensity was reduced, until the participants indicated that the pain had disappeared, and that value was noted. It was increased again until the pain reappeared, and the corresponding intensity value was recorded. The maximum value of the three readings was recorded as the first pain threshold [38]. The currents were administered under a compliance of 400 V, with a Pulse Duration of 2000 m. The intensity was always increased and decreased in ranges of 0.05 mA. In the search for the sensory threshold, the current amplitude ×1 was used, and to determine the first pain threshold, the current amplitude ×10 was used.

(IV) Blood test

A venous blood sample was collected from the antecubital vein. After centrifugation, the sample was transported to the laboratory for immediate analysis. Blood samples were always collected prior to the physical tests. Serum samples were processed using the Architect c-8000 system (Abbott Laboratories, 100 Abbott Park Road, Abbott Park, IL, USA) using the chemiluminescence method for the determination of LH, FSH, 17B-Estradiol, progesterone, SHBG, and testosterone.

(V) Warm-up and fitness level assessment tests

After this, the participants performed a standardized 5 min stationary bike warm-up, between 75 and 85 rpm (Cateye EC-3200, Osaka, Japan). Subsequently, all the athletes performed a protocol of 5 exercises for the warm-up of muscle groups of legs, trunk, and arms.

Subsequently, the athletes proceeded with a battery of physical tests to assess flexibility, hand grip strength, high-speed strength, and cardiorespiratory fitness. There were a total of 8 tests in the following order:

- The sit and reach test was used to measure flexibility [39].
- Maximum grip dominant hand (hand grip) [40,41] was measured with a dynamometer (Takei TKK 5101, Tokyo, Japan). The athletes were instructed to stay in a relaxed position, and after a countdown of 3 s, they had to make the maximum possible force with their hand on the dynamometer, maintaining the maximum prehensile force for 5 s. Previously, the width of the dynamometer was adjusted to the comfort of each athlete. Jump battery [42]: squat jump with additional weight (50% body weight) [43], squat jump without additional weight [44], counter movement jump [44], Abalakov jump [43], drop jump from 40 cm with arm swing) [43,45,46]. The jump height was estimated by the flight time measured with a contact platform (Chronojump, Barcelona, Spain) [47]. Before the jumps, they were instructed on how to perform each of them. For squat jump with additional weight, the participant placed a bar over the shoulders with the corresponding weight.
- Finally, the Course Navette test [48]. The test was stopped by the participant’s own fatigue or when for 2 consecutive times they failed to step on the line that marks the 20 m before the test sounds. The measurement of maximum oxygen consumption ($\dot{V}$O_2_max) was carried out. The measurement of maximum oxygen consumption ($\dot{V}$O_2_max) was indirectly determined using the Course Navette test, following the protocol developed by Luc Léger et al. [49]. The predicted $\dot{V}$O_2_max was calculated using the following formula: predicted $\dot{V}$O_2_max = −24.4 + 6 × X, where X_1_ represents the athlete’s maximal shuttle speed (km/h). Additionally, the distance covered by the athletes during the test was measured using the method described by Garcia and Secchi [50].

For these tests (excluding the Course Navette test) all participants made three attempts with a one-minute rest between each attempt. The best result obtained was recorded.

#### 2.2.4. Statistical Analysis

The sample size was calculated based on a previous study [51] which reported a mean VO_2_ of 4.49 (SD = 0.98; IC 95% [3.911,5.069]) in phase I and 4.52 (SD = 0.90; IC 95% [3.988,5.052]) in phase III. Considering the correlation values between VO_2_ (r = 0.83) as reported by Farrell et al. [52] and aiming for a statistical power of 85%, a sample size of 46 subjects was estimated to detect a mean difference of 0.25 VO_2_ (ml·kg^−1^·min^−1^) between the follicular and luteal phases.

Statistical analyses were performed using the Statistical Package for the Social Sciences (IBM SPSS Statistics for Windows, version 25.0, IBM Corp., Armonk, NY, USA), and values of *p* < 0.05 were considered statistically significant. The normal distribution of variables was verified by the Shapiro–Wilk test. As the variables were not normally distributed, non-parametric statistical tests were applied. To describe the collected data, we used the mean and standard deviation for continuous variables and frequency for categorical variables. The Friedman test was used to analyze the evolution of parameters throughout the natural MC, and the Wilcoxon test was used for the evaluation of contraceptive MC parameters. Post hoc comparisons were made using the Bonferroni adjustment for multiple comparisons. The Mann–Whitney U test was used to compare parameters between the two study groups. The effect size was determined using Cohen’s d. A d value of 0.1 indicated a very small effect size, d (0.2) small, d (0.5) medium, d (0.8) large, d (1.2) very large, and d (2.0) huge [53,54].

Given the limited sample size and the non-normal distribution of the independent variables, the residual errors of the resulting models were inspected to ensure their normal distribution and thus the reliability of the regression models. To determine the predictive value of the model, the Cohen criteria were applied to unidirectional ANOVA models. This criterion indicates that the values of R^2^ below 0.10 do not give a relevant explanatory value; an R^2^ between 0.10 and 0.25 indicates a dependence of the analyzed variables on the explanatory variance of the identified factors; and R^2^ values greater than 0.25 allow us to affirm that the explanatory model is clinically relevant.

## 3. Results

A total of 41 women (natural MC, *n* = 27; contraceptive MC, *n* = 14) were part of the research. After analyzing the blood tests’ results, data from a total of 34 women were included in the analysis (women with natural MC, *n* = 20, and women with contraceptive MC, *n* = 14). Seven women with natural MC were excluded from the analyses for not having a progesterone greater than 16 nmol·L^−1^ during the luteal phase [17,23]. Figure 2 shows a flow chart regarding the recruitment process and inclusion of participants.

Regarding the characteristics of the participants, they are shown in Table 1. No statistically significant differences were found between the two study groups.

### 3.1. Sex Hormones

Table 2 shows the levels of natural MC sex hormones in the different phases analyzed. Progesterone, estrogen, P/E ratio, FSH, LH, total testosterone, and FAI levels differed between phases.

Table 3 shows the sex hormone levels of the contraceptive MC. Levels of estrogen, P/E ratio, FSH, LH, total testosterone, SHBG, and FAI show significant differences between the inactive HC phase and the active phase.

### 3.2. Fitness Level and MC Phases

Table 4 shows the variables related to the level of physical fitness of the women with a natural MC. The meters traveled in the Course Navette test show differences in phase I compared to phase II (*p* = 0.005; d = 1.15) and phase III (*p* = 0.034; d = 0.40). The $\dot{V}$O_2_max shows differences in phase I compared to phase II (*p* = 0.004/d = 1.45) and phase III (*p* = 0.043/d = 0.49). The high-speed strength shows differences in the Abalakov jump between phase I and phase II (*p* = 0.001; d = 0.71).

Table 5 shows the variables related to the level of physical fitness of the women with a contraceptive MC. The meters traveled in the Course Navette test (*p* = 0.040; d = 0.59), flexibility (*p* = 0.041; d = 0.22) and high-speed strength by SJ with 50% additional weight (*p* = 0.009; d = 0.40), differed between the inactive phase and active phase of the contraceptive menstrual cycling.

### 3.3. Body Composition

Table 4 shows the information related to the body composition of natural MC. The weight differed between phase I and phase III (*p* = 0.006; d = 0.60), and the same occurred with the percentage of fat mass (*p* = 0.011; d = 0.54). The BMI showed variation between phase I and II (*p* = 0.003; d = 1.15) and between phase I and III (*p* = 0.040; d = 0.68).

Table 5 shows the information related to the body composition of the contraceptive MC. As in natural MC, the variables that differ between the two key phases of contraceptive MC are weight (*p* = 0.027; d = 0.87), BMI (*p* = 0.017; d = 0.66), and percentage of fat mass (*p* = 0.014; d = 0.76).

### 3.4. Sensory Threshold and Pain Threshold

Regarding natural MC, Table 4 shows information related to the sensory and first pain thresholds. In the first pain threshold, there are significant differences between phase I and II (*p* = 0.027; d = 0.40) and between phase I and III (*p* = 0.011; d = 0.31); however, these differences are not evident in contraceptive MC (Table 5). Significant differences were also found in sensory threshold in the natural MC between phase I and II (*p* = 0.017; d = 0.50).

#### Multiple Regression Analysis

The results of the multiple regression analysis are as follows in Table 6. When performing the multiple linear regression analysis using $\dot{V}$O_2_max as a dependent variable, we found that in phase I of athletes with natural MC, the first pain threshold justifies 31.9% of the variance, which we consider as clinically relevant if we follow Cohen’s criteria [53,54].

## 4. Discussion

The objective of the present study was to observe whether the MC, both natural MC and contraceptive cycling, causes variations related to the level of physical fitness, body composition, pain, and sensitivity. The latest recommendations of the literature on how to properly verify the phases of the MC are followed and the results of women who did not obtain a minimum value of progesterone to consider the natural MC as ovulatory are eliminated from the analysis [17]. Therefore, it is expected that the following lines will be satisfactory to draw conclusions about the MC and its relationship with the physical fitness of athletes.

### 4.1. Influence of Natural MC on Cardiorespiratory Fitness

The main finding of the present study was the variation of $\dot{V}$O_2_max and the meters covered in the Course Navette test between the phases of the natural MC. Although some studies have concluded that no changes have been found in $\dot{V}$O_2_max related to MC [11,55], this may be due to a low number of participants (*n* < 10), the potential inclusion of deficient luteal phases due to the lack of hormonal blood concentration determination, or the monitoring of <= 2 phases of MC. In this research, both $\dot{V}$O_2_max and the meters covered in the Course Navette test were significantly lower in phase I compared to phases II and III. In line with our research, Barba-Moreno et al. [11,56] observed a lower $\dot{V}$O_2_max in phase I.

The variation in $\dot{V}$O_2_max could be attributed to the significant changes found in sex hormone levels between phases. In women, estrogens have been positively linked to physical fitness [7]. In phase I (140.39 pmol/L), estrogens are significantly lower than phase II and III (493.34 and 519.26 pmol/L) (*p* < 0.05), so it could justify the variation of $\dot{V}$O_2_max. Estrogens cause anabolic effects, increasing glycogen uptake and storage [57,58]. In addition to estrogen, testosterone has a powerful effect on muscle tissue [59,60] and has been attributed a protective role against severe fatigue [61], and this variable is significantly higher in phase II compared to other phases. Testosterone levels followed normative serum androgen values in elite female athletes determined by Stéphane Bermon et al. [18].

Another important discovery was that weight, BMI, and fat percentage were higher in phase I of natural MC compared to phase III. The percentage of water and muscle mass did not change, suggesting that weight difference is associated with the percentage of fat. Coinciding with this observation, the $\dot{V}$O_2_max was lower in phase I compared to phase III. It could be said that the achievement of the $\dot{V}$O_2_max is related to the weight and % of fat of the athlete, and these were greater in phase I, as the women did not reach the maximum levels as achieved in the rest of the phases. $\dot{V}$O_2_max is expressed in mL·kg^- 1^·min^−1^, so it makes sense that changes in body weight can influence it [15]. In line with our results, Martínez-Navarro et al. [62] confirmed that women’s % fat influences the $\dot{V}$O_2_max variable.

### 4.2. Influence of Natural MC on High-Speed Strength, Hand Grip Strength, and Flexibility

Regarding the level of hand grip strength, in line with various studies, the hand grip of the dominant hand does not differ between the phases of the natural MC [63,64].

Most tests related to lower body high-speed strength were not significant, in line with a study that analyzed the CMJ in soccer players [10]. However, this study only studied this variable in phase I and III. As Thompson et al. already indicated in their study [63], it is important to take into account the late follicular phase (phase II in this research) when estrogen is high and progesterone low, where several studies have confirmed that there is a decrease in musculotendinous stiffness of the lower extremities [65] and an increase in muscle extensibility and joint laxity [66], being detrimental to the jump, because flexible musculature reduces the development of high-speed strength. Thompson et al. [63] stated that the faster and more explosive aspects of muscle performance could be influenced by MC hormones. In our study, the height in centimeters for the Abalakov jump was significantly shorter during phase II than during phase I. No other jumps showed significant differences, on the contrary, studies that analyze the CMJ jump in women who perform physical activity recreationally [63,64] have found differences between the phases. These controversial results could be due to the difference in the baseline physical fitness level of the participants, because in studies that do not show differences, participants are female athletes. This must be due to the technical component of the Abalakov jump, as the arms are used, unlike in the other jumps, and it is most frequently used among female athletes.

Regarding flexibility, no significant differences were reported. However, Campa et al. [67] did find differences between phase I and phase II of the menstrual cycle. The discrepancies in findings could be attributed to differences in verifying the phases or variations in sample characteristics. Our results, where flexibility remains unchanged, align with the findings on high-speed strength in our study. If flexibility does not vary, muscle–tendon stiffness and muscle extensibility would remain constant, not affecting the results of the jumps. Therefore, it is believed that estrogen fluctuation is not capable of significantly influencing flexibility. Similarly, it is believed that estrogens do not have a significant impact on high-speed strength and hand grip strength. As evidenced by the measurement of estrogen in this research, high-speed strength and hand grip strength levels could be expected to be higher in phases II and III compared to phase I. However, the finding regarding these variables make us reject the hypothesis that estrogens increase the level of high-speed strength, because this is when these are lower. This finding is consistent with the study conducted by Tine Vrist Dam et al. [64], who also rejected this hypothesis. It is thought that the fluctuation of estrogen associated with the menstrual cycle does not cause substantial changes in high-speed and hand grip strength.

### 4.3. Influence of Natural MC on the Pain and Sensory Thresholds

Another important finding was related to sensory threshold and first pain threshold. M. Sambanis et al. [68] in their research confirmed that female athletes felt increased fatigue and weakness during menstruation (phase I). In our study, the sensory threshold was lower in phase I compared to phase II, and the first pain threshold was lower in phase I compared to phases II and III, and consequently, we could affirm that women have a greater sensitivity and a lower tolerance to pain in the first phase of the cycle. We consider that this finding could be related to the hormonal fluctuations experienced during the MC. Therefore, the decrease in this threshold could be related to the values of $\dot{V}$O_2_max. The first pain threshold was significantly higher in phases II and III, so they could help female athletes achieve higher levels of cardiorespiratory fitness. These results are reaffirmed by the association found through regression analyses between $\dot{V}$O_2_max and first pain threshold in phase I, explaining 31.9% of the variance. Romero-Parra et al. [69] confirmed that women perceived muscle soreness more severely before exercise performed in phase I, when estrogen concentrations are relatively low, which may affect women’s predisposition to strenuous exercise during this phase.

The methodological approach used in this research, with the incorporation of the measurement of pain and sensitivity related to the phases of MC, is novel in the scientific literature, because no similar work has been found, where a combined measurement of both variables occurs. With the results obtained, we consider it pertinent to open new lines of research in this direction.

### 4.4. Influence of Contraceptive Menstrual Cycling Phases on the Level of Cardiorespiratory Fitness, High-Speed Strength, Hand Grip Strength, and Flexibility

In our research, significant differences have been found between some study variables for contraceptive MC. Conversely, a systematic review on the influence of the contraceptive MC on performance concluded that there were no differences between the phases, but 83% of its articles were rated as moderate, low, or very low methodological quality [4]. In this study, differences have been found on the meters traveled in the Course Navette test. As in the natural MC, the weight and % of fat differs significantly between phases and may be associated with the variation of meters reached in the Course Navette test, because women travel more meters when the % of fat is lower, and although the change is small, it is considered significant in athletes. Unlike in the contraceptive MC, there was not much difference in the $\dot{V}$O_2_max, only on the meters traveled. Similarly, Barba-Moreno et al. [56] also found no difference in maximal oxygen consumption. In addition to the above explanation, the fact that the $\dot{V}$O_2_max fluctuates is associated with the high level of estrogens, and although these vary significantly between the inactive phase and the active phase, they are not high enough levels to cause changes in this variable. Therefore, we can confirm that in our study the hormonal fluctuation related to the contraceptive MC does not cause substantial changes in the cardiorespiratory fitness.

As far as the measurement of the level of force is concerned, the hand grip does not differ in this type of cycle [63,64] either. Regarding the jumps, there are differences in the squat jump with additional weight. It is thought that the significant variation in weight and % of fat between phases has contributed to the athletes performing better in the active phase, compared to the inactive contraceptive phase where the weight was higher. This jump involves moving 50% of your body weight; therefore, in addition to decreasing body weight in this phase, the additional load is also decreased, which may be the reason for the increase in centimeters in this jump. Regarding flexibility, despite statistically significant differences between both phases, the *p*-value and effect size are small (*p* = 0.041; d = 0.22), indicating that this association requires further investigation.

### 4.5. Study Limitations

Although this research consisted of 41 participants (a total of 123 assessments), it is considered more appropriate to increase the sample size following the recommendations of the literature [23]. In the future, a comparison between the study groups could be made, in order to clarify whether or not there is a difference in the physical fitness between both types of cycles. In addition, future research to study women with deficient luteal phases and their characteristics related to sports performance would be useful.

Another limitation encountered in this study is the complexity associated with monitoring this type of assessment among athletes classified between levels II and III of McKay’s classification [33]. It presents a challenge to schedule visits according to each woman’s menstrual cycle and accommodate their sports routines. Circadian rhythm, nutrition, and prior rest are influential factors on performance that have not been controlled for in the current study. The participants were allowed to have less strict prior fasting and shorter rest between training sessions, which are difficult-to-control factors [70].

## 5. Conclusions

Athletes were classified between levels II and III of McKay’s classification, have a natural menstrual cycle, and experience changes in $\dot{V}$O_2_max depending on the phase of the MC they are in. The pain and sensory thresholds fluctuate between the first two phases and seem to affect the $\dot{V}$O_2_max.

Athletes with contraceptive MC experience differences in the meters traveled in the Course Navette test and in the squat jump with additional weight and it seems to be related to the fluctuation of body weight that also occurs in this type of cycle.

Based on the results obtained, this article recommends adapting training and competitions for athletes classified as level II and III according to McKay’s classification.

In athletes with contraceptive MC, there is no variation in the sensory and first pain thresholds or in the $\dot{V}$O_2_max. Neither do the women benefit from the increase in the cardiorespiratory fitness in phases II and III found in women with natural MC, so the use of HC is not harmless with respect to their physical condition.

It highlights the need for the correct verification of the phases of the MC by blood measurement of sex hormones, so that the correct scientific conclusions can be drawn.

## Figures and Tables

**Table 1:** Participant characteristics.

Measurement	Natural MC (*n* = 20)	Contraceptive MC (*n* = 14)	*p* Value
Age (year)	26.55 ± 5.880	26.86 ± 5.187	0.769
Height (cm)	165.21 ± 6.529	162.11 ± 5.088	0.231
BMI (kg·m^−2^)	23.06 ± 2.419	22.79 ± 3.087	0.545
Age at first menstruation (year)	12.15 ± 1.137	12.62 ± 1.557	0.501
Duration of cycles	27.90 ± 2.732	27.23 ± 1.739	0.598
Duration of bleedings	4.41 ± 0.795	3.83 ± 0.937	0.166
Years practicing sport	13.75 ± 8.22	14.14 ± 8.55	0.890

Values are presented as mean ± SD. The data shown refer to phase I of both groups.

**Table 2:** Sex hormone levels in the natural menstrual cycle.

Measurement Natural Cycle	PHASE I	*p* Value/d de Cohen	PHASE II	*p* Value/d de Cohen	PHASE III	*p* Value/d de Cohen
Progesterone (nmol/L)	1.98 ± 1.09 (1.47 to 2.49) ^b^	0.022/0.80	9.21 ± 8.84 (5.07 to 13.35) ^c^	0.005/1.66	33.89 ± 9.80 (29.30 to 38.48) ^a^	0.001/3.28
Estrogen (pmol/L)	140.39 ± 84.50 (100.84 to 179.94) ^b^	0.000/1.05	493.34 ± 326.31 (340.62 to 646.06)		519.26 ± 192.54 (429.14 to 609.38) ^a^	0.001/2.05
P/E ratio	20.94 ± 19.87 (26.84–9.59) ^b^		29.07 ± 29.52 (39.73–4.75) ^c^	0.000/1.01	72.20 ± 29.30 (94.79–50.64) ^a^	0.001/2.16
FSH (mIU/mL)	6.01 ± 1.69 (5.21 to 6.80) ^b^		5.88 ± 3.53 (4.22 to 7.54) ^c^	0.000/0.83	2.75 ± 0.90 (2.32 to 3.17) ^a^	0.001/1.79
LH (mIU/mL)	4.07 ± 1.83 (3.21 to 4.92) ^b^	0.000/0.61	15.07 ± 18.10 (6.59 to 23.54) ^c^	0.000/0.62	3.80 ± 2.41 (2.67 to 4.93)	
Total testosterone (nmol/L)	1.21 ± 0.37 (1.03 to 1.38) ^b^	0.02/0.54	1.38 ± 0.41 (1.18 to 1.57) ^c^	0.02/0.84	1.13 ± 0.24 (1.01 to 1.24)	
SHBG (nmol/L)	75.69 (63.41 to 87.96)		81.13 (66.32 to 95.93)		83.98 (68.12 to 99.84)	
Free androgen index (nmol/L)	1.68 ± 26.22 (1.45 to 1.91)		1.92 ± 31.63 (1.49 to 2.35) ^c^	0.011/0.46	1.54 ± 33.88 (1.22 to 1.86) ^a^	0.027/0.30

Values are presented as mean ± SD and 95% confidence interval. Only statistically significant *p*-values are shown; ^a^ Significantly different from phase I; ^b^ Significantly different from phase II; ^c^ Significantly different from phase III; P/E ratio: progesterone/estrogen ratio; free androgen index: [(total testosterone/SHBG) × 100].

**Table 3:** Sex hormone levels in the contraceptive menstrual cycling.

Measurement Contraceptive MC	Inactive HC Phase	Active HC Phase	*p* Value	d de Cohen
Progesterone (nmol/L)	2.22 ± 0.95 (1.67 to 2.77)	2.95 ± 1.78 (1.60 to 3.60))	0.470	0.28
Estrogen (pmol/L)	103.14 ± 130.53 (27.77 to 178.51)	30.05 ± 58.29 (−3.60 to 63.71)	0.016	0.49
P/E ratio	63.90 ± 79.03 (18.27 to 109.54)	200.44 ± 211.18 (78.50 to 322.37)	0.026	0.70
FSH (mIU/mL)	5.05 ± 3.42 (3.33 to 7.29)	1.07 ± 1.27 (0.34 to 1.81)	0.001	1.2
LH (mIU/mL)	2.72 ± 2.41 (1.33 to 4.12)	0.59 ± 0.02 (0.06 to 1.12)	0.003	0.8
Total testosterone (nmol/L)	1.34 ± 0.41 (1.10 to 1.58)	1.01 ± 0.25 (0.86 to 1.16)	0.003	1.09
SHBG (nmol/L)	217.91 ± 92.81 (164.32 to 271.50)	361.59 ± 142.64 (279.23 to 443.95)	0.002	1.51
Free androgen index (nmol/L)	0.84 ± 0.76 (0.39 to 1.28)	0.36 ± 0.24 (0.22 to 0.50)	0.005	0.60

Values are presented as mean ± SD and 95% confidence interval. Only statistically significant *p*-values are shown; P/E ratio: progesterone/estrogen ratio; free androgen index: [(total testosterone/SHBG) × 100].

**Table 4:** Association of the phases of the natural MC and parameters of sports performance, bioimpedance, and sensory and first pain thresholds.

Measurement Natural Cycle	PHASE I	*p* Value/d de Cohen (I vs. II)	PHASE II	*p* Value/d de Cohen (II vs. III)	PHASE III	*p* Value/d de Cohen (I vs. III)
Body mass (kg)	63.23 ± 10.05 (58.52 to 67.94) ^c^		62.64 ± 9.66 (58.11 to 67.16)		62.48 ± 9.74 (57.92 to 67.04) ^a^	0.006/0.60
Total body water (L)	36.50 ± 5.02 (34.15 to 38.84)		36.39 ± 4.90 (34.09 to 38.68)		36.42 ± 4.66 (34.23 to 38.60)	
Body fat percent (%)	20.61 ± 6.691 (17.48 to 23.74) ^c^		20.18 ± 6.603 (17.09 to 23.27)		19.85 ± 6.583 (16.76 to 22.93) ^a^	0.011/0.54
Skeletal muscle mass	27.84 ± 4.06 (25.93 to 29.73)		27.75 ± 3.9 (25.87 to 29.62)		27.82 ± 3.86 (26.01 to 29.62)	
BMI (kg·m^−2^)	23.05 ± 2.41 (21.92 to 24.18) ^b,c^	0.003/1.15	22.84 ± 2.35 (21.73 to 23.94) ^a^		22.79 ± 2.37 (21.67 to 23.90) ^a^	0.040/0.68
Course Navette (m)	1100 ± 332.96 (944 to 1255) ^b,c^	0.005/1.15	1207 ± 316.91 (1058 to 1355) ^a^		1176 ± 396.91 (990 to 1361) ^a^	0.034/0.40
$\dot{V}$O_2_max (mL·kg^−1^·min^−1^)	41.75 ± 5.28 (39.27 to 44.22) ^b,c^	0.004/1.45	43.85 ± 5.13 (41.44 to 46.25) ^a^		43.25 ± 6.19 (40.35 to 46.14) ^a^	0.043/0.49
Hand grip dominant hand (Kg)	32.15 ± 6.93 (28.90 to 35.40)		32.87 ± 7.80 (29.22 to 36.52)		33.51 ± 6.53 (30.45 to 36.56)	
Sit and reach (cm)	11.15 ± 7.41 (7.68 to 14.62)		11.28 ± 7.38 (7.82 to 14.74)		11.97 ± 6.70 (8.83 to 15.11)	
SJ 50% additional body weight (cm)	14.08 ± 3.74 (12.33 to 15.84)		14.40 ± 3.76 (12.67 to 16.20)		14.85 ± 4.04 (12.96 to 16.74)	
SJ (cm)	26.49 ± 5.19 (24.06 to 28.92)		25.97 ± 5.52 (23.39 to 28.56)		26.74 ± 5.91 (23.74 to 29.51)	
CMJ (cm)	27.80 ± 5.40 (25.27 to 30.33)		27.38 ± 5.00 (27.38 to 29.73)		28.58 ± 6.10 (25.72 to 31.44)	
ABK jump (cm)	30.23 ± 5.19 (29.28 to 34.63) ^b^	0.001/0.71	29.15 ± 5.45 (27.77 to 32.77) ^a^		30.21 ± 6.00 (28.47 to 34.87)	
DJ 40 cm (cm)	24.32 ± 6.25 (21.39 to 27.24)		25.39 ± 6.42 (22.38 to 28.40)		25.45 ± 7.72 (21.84 to 29.07)	
Sensory threshold (mA)	0.64 ± 0.22 (0.53 to 0.74) ^b^	0.017/0.50	0.76 ± 0.29 (0.62 to 0.89) ^a^		0.75 ± 0.29 (0.61 to 0.89)	
First pain threshold (mA)	1.34 ± 1.05 (0.85 to 1.83) ^b,c^	0.027/0.40	1.69 ± 1.60 (0.94 to 2.44) ^a^		1.59 ± 1.31 (0.97 to 2.20) ^a^	0.011/0.31

Values are presented as mean ± SD and 95% confidence interval. Only statistically significant *p*-values are shown; ^a^ Significantly different from phase I; ^b^ Significantly different from phase II; ^c^ Significantly different from phase III; Only *p*-values and Cohen’s d values *p* < 0.05 are presented. SJ: squat jump, CMJ: counter movement jump, ABK jump: Abalakov jump; DJ: drop jump.

**Table 5:** Association of the phases of the contraceptive MC and parameters of sports performance, bioimpedance, and sensory and first pain thresholds.

Measurement Contraceptive Menstrual Cycling	Inactive HC Phase	Active HC Phase	*p* Value
Body mass (kg)	59.75 ± 7.67 (55.32 to 64.18)	59.02 ± 7.68 (54.58 to 63.45)	0.027/0.87
Total body water (L)	33.49 ± 2.70 (31.93 to 35.05)	33.56 ± 2.54 (32.08 to 35.02)	
Body fat percent (%)	22.64 ± 7.88 (18.08 to 27.19)	21.54 ± 7.96 (16.94 to 26.14)	0.014/0.76
Skeletal muscle mass	25.34 ± 2.23 (24.04 to 26.62)	25.39 ± 2.10 (24.17 to 26.60)	
BMI (kg·m^−2^)	22.79 ± 3.08 (21.00 to 24.56)	22.49 ± 3.04 (20.73 to 24.25)	0.017/0.66
Course Navette (m)	1110.00 ± 305.81 (933 to 1285)	1185.71 ± 307.58 (1008 to 1363)	0.040/0.59
$\dot{V}$O_2_max (mL·kg^−1^·min^−1^)	42.02 ± 4.53 (39.41 to 44.64)	42.60 ± 6.34 (38.93 to 46.26)	
Hand grip hand dominant (kg)	27.82 ± 3.32 (25.89 to 29.74)	29.31 ± 3.89 (27.06 to 31.56)	
Seat and reach (cm)	10.95 ± 6.68 (7.09 to 14.81)	11.51 ± 7.68 (7.07 to 15.95)	0.041/0.22
SJ WITH 50% additional body weight (cms)	12.15 ± 5.84 (8.77 to 15.52)	13.48 ± 5.85 (10.10 to 16.86)	0.009/0.40
SJ (cms)	23.26 ± 6.81 (19.32 to 27.19)	23.80 ± 7.29 (19.58 to 28.01)	
CMJ (cms)	24.20 ± 7.10 (20.10 to 28.31)	25.07 ± 7.83 (20.55 to 29.59)	
ABK jump (cms)	27.71 ± 8.03 (23.07 to 32.35)	28.52 ± 8.05 (23.88 to 33.17)	
DJ (cms)	23.42 ± 9.87 (17.17 to 29.12)	22.02 ± 8.77 (16.95 to 27.09)	
Sensory threshold (mA)	0.57 ± 0.26 (0.42 to 0.73)	0.62 ± 0.19 (0.51 to 0.73)	
First pain threshold (mA)	1.43 ± 0.98 (0.86 to 2.00)	1.67 ± 1.14 (1.01 to 2.33)	

Values are presented as mean ± SD and 95% confidence interval. Only statistically significant *p*-values are shown; Only *p*-values and Cohen’s d values *p* < 0.05 are presented. SJ: squat jump, CMJ: counter movement jump, ABK jump: Abalakov jump; DJ: drop jump.

**Table 6:** Result of the multiple regression analysis.

Model	R^2^ Adjusted	Standardized Coefficients Beta	Standard Error	F (*p*)
Dependent Variable: $\dot{V}$O_2_max Covariates: First pain threshold	0.319	0.595	4.3619	9.885 (0.006)

## Data Availability

The data presented in this study are available on request from the corresponding author.
